# A Modular Microfluidic Device *via* Multimaterial 3D Printing for Emulsion Generation

**DOI:** 10.1038/s41598-018-22756-1

**Published:** 2018-03-19

**Authors:** Qinglei Ji, Jia Ming Zhang, Ying Liu, Xiying Li, Pengyu Lv, Dongping Jin, Huiling Duan

**Affiliations:** 1 State Key Laboratory of Mechanics and Control of Mechanical Structures, Nanjing University of Aeronautics and Astronautics, 29 Yudao Street, Nanjing, 210016 Jiangsu People’s Republic of China; 2 State Key Laboratory for Turbulence and Complex Systems, Department of Mechanics and Engineering Science, BIC-ESAT, College of Engineering, Peking University, Beijing, 100871 People’s Republic of China; 3 CAPT, HEDPS and IFSA Collaborative Innovation Center of MoE, Peking University, Beijing, 100871 People’s Republic of China

## Abstract

3D-printing (3DP) technology has been developing rapidly. However, limited studies on the contribution of 3DP technology, especially multimaterial 3DP technology, to droplet-microfluidics have been reported. In this paper, multimaterial 3D-printed devices for the pneumatic control of emulsion generation have been reported. A 3D coaxial flexible channel with other rigid structures has been designed and printed monolithically. Numerical and experimental studies have demonstrated that this flexible channel can be excited by the air pressure and then deform in a controllable way, which can provide the active control of droplet generation. Furthermore, a novel modular microfluidic device for double emulsion generation has been designed and fabricated, which consists of three modules: function module, T-junction module, and co-flow module. The function module can be replaced by (1) Single-inlet module, (2) Pneumatic Control Unit (PCU) module and (3) Dual-inlet module. Different modules can be easily assembled for different double emulsion production. By using the PCU module, double emulsions with different number of inner droplets have been successfully produced without complicated operation of flow rates of different phases. By using single and dual inlet module, various double emulsions with different number of encapsulated droplets or encapsulated droplets with different compositions have been successfully produced, respectively.

## Introduction

Droplet microfluidics has been developed for the generation and manipulation of monodisperse droplets and bubbles in a continuous flow^1,2^. It has attracted more and more attentions due to its extensive applications in biology^3,4^, chemistry^5^ and nanotechnology^6,7^. Single emulsions have been first studied for fast analytical systems^8^ and the synthesis of advanced materials^9^. More complicated multiple emulsions have been further developed for the controlled encapsulation and release of materials in cosmetic^10^, drug delivery^11^ and food applications^12^.

The prerequisite consideration in droplet microfluidics is the generation of emulsion droplets. One common method for fabricating emulsion generator is soft-lithography^13^, which has been widely applied due to its capability of fabricating different microchannels, such as T-junction^14^, flow-focusing^15^ and co-flow^16^, with high resolution down to 1 μm. However, it includes relatively complicated fabrication process and normally requires expensive master molds and clean room environment, which limit its wide usage^17^. The other popular method is using glass capillaries^18–20^. Glass capillaries are welcomed due to its excellent optical transparency, electrical insulativity and chemical robustness. Even higher-order multiple emulsions can be achieved through assembly of glass capillaries^21^. Nevertheless, glass capillary devices require manual operation, which may cause instability and inconsistency issues. In addition, it is difficult to manufacture complex three-dimensional (3D) structures using these methods. To make extensive applications of droplet-microfluidics, it is essential to develop new techniques for emulsion generation in a simple, low-cost and reliable manner.

In recent years, typical 3D-printing (3DP) technologies such as fused deposition modeling (FDM) and stereolithography (SL) have been applied in bioengineering^22–26^ and starts to find a place in the microfluidic field^27–29^. Micromixer^30,31^, droplet generator^32,33^, reactionware^34,35^, helical channel^36^, check valve^37,38^ and pump^39^ manufactured *via* such typical 3DP technologies have been reported. Besides typical 3DP technologies, novel 3DP methods are still continuously emerged to create more possibilities for the academic community^40,41^. More recently, novel multimaterial 3DP has been developed and benefits various fields such as synthesis of novel functional materials^42,43^, creation of heterogeneous organ-on-a-chip^44^ and fabrication of novel microfluidic devices. One of the greatest advantages of multimaterial 3DP is that versatile materials can be simultaneously used for building a single object, which is ideal for fabrication of microfluidic control components consisting of different parts such as interconnects^45^, membranes^46,47^, valves and pumps^48–50^, and multi-flow controllers^51^. Compared to traditional methods, 3DP technology has distinct advantages and disadvantages for microfluidics. It offers a new rapid-prototyping and 3D-digital manufacturing method which can fabricate microfluidic devices in a simple, fast, customized and monolithic manner. On the other hand, the resolution of printing, the biocompatibility of printed materials, as well as the optical property and surface property of printed materials are still unsatisfying for the microfluidic community. Potentials of 3DP for more feature-rich microfluidic devices still need to be exploited.

Following the mechanical control methods for emulsion generation^52,53^, pneumatic control of generation of single and double emulsions in modular 3D-printed devices has been reported in the present study. A novel pneumatic control unit (PCU) has been designed and fabricated monolithically by multimaterial 3DP technology. This PCU has also been numerically and experimentally studied for its effects on single emulsion generation, and then we extend its capacity in active control of the generation of double emulsions with different number of encapsulated droplets without complicated operation of flow rates of different phases. Furthermore, modular 3D-printed devices for generation of various double emulsions and microspheres have been reported, which has demonstrated that 3D-printed devices can be used for the complicated operation of multiple emulsions. Potentials of 3DP technology in droplet-microfluidic fields, especially for generation of multiple emulsions, have been exploited.

## Materials and Methods

**Figure 1:**
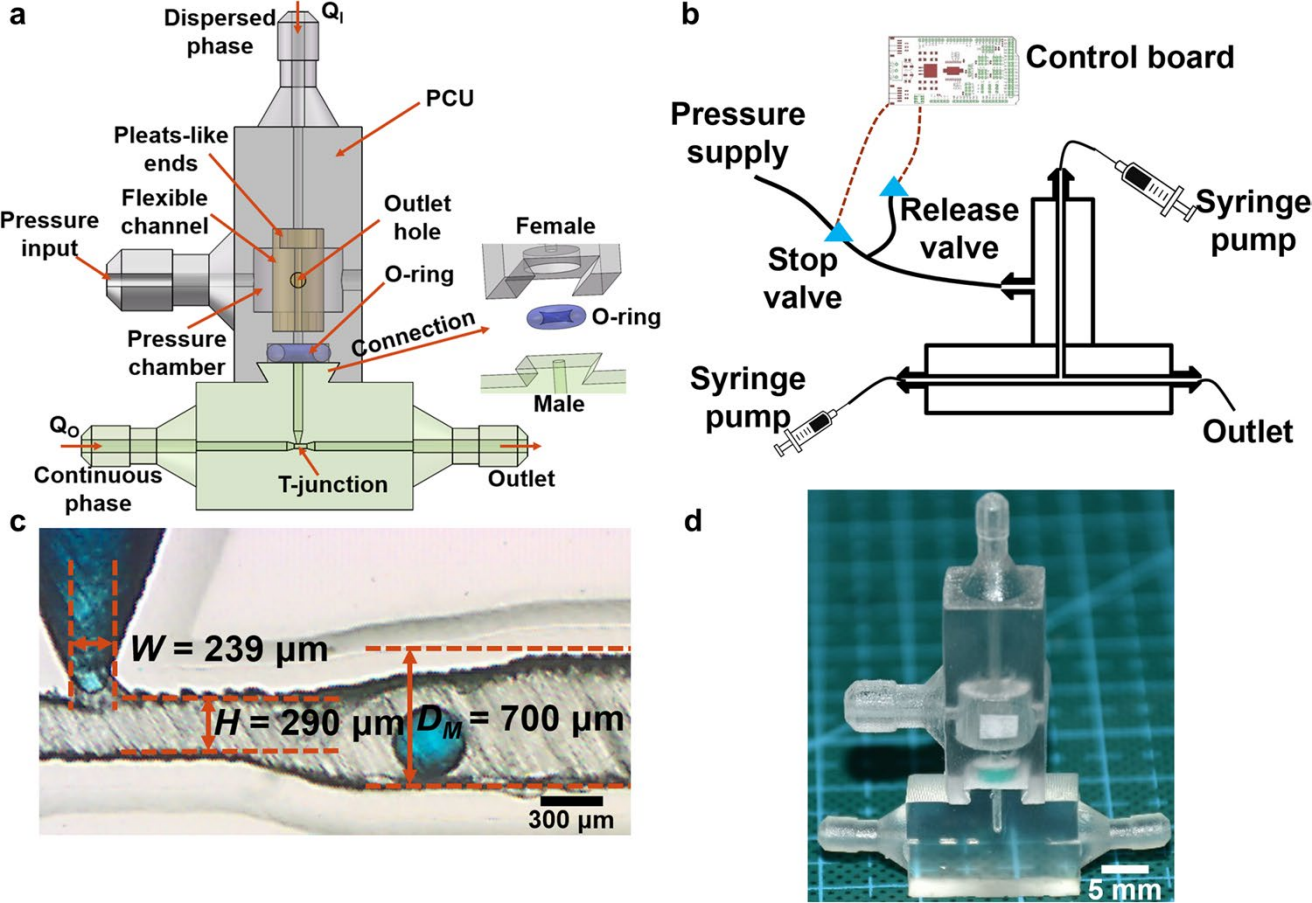
(**a**) Schematic of the pneumatic device for droplet generation. (**b**) Schematic of the experimental setup. (**c**) Photograph showing T-junction channel. (**d**) A printed pneumatic device for emulsion generation.

All printed files were designed using Solidworks (Dassault Systèmes). As shown in Fig. 1a, a single emulsion generator mainly includes two parts, PCU and droplet generator (T-junction). The PCU was printed with a multimaterial 3D-printer, Objet 350 Connex 3 (Stratasys, Ltd.). Veroclear, a transparent PolyJet photopolymer provided by the same company, was used to print the rigid part (grey). A rubber-like material, TangoPlus FLX930 was used to print the 3D coaxial flexible channel (orange). The flexible channel has pleat-like ends (See supplementary Fig. S1), which makes adhesion to the rigid part with larger areas to increase the structure strength. The flexible channel is located in a pressure chamber, which is connected with a pneumatic pump (AT60/25, Jiangsu dynamic medical technology Ltd.) to excite this flexible channel. Water solution containing 2% NaOH (w/w) and 1% Na_2_SiO_3_ (w/w) was used for dissolution of support materials, and then the printed PCU was put in an ultrasonic device for 2 hours to fully remove the support materials. Since our PCU structure is relatively simple and small, the required printing time is about 20 minutes, and 2 hours for removal of supports are enough. It will take longer time to print and remove supports if the structure to print is complicated.

The T-junction structure (green) was printed using a stereolithography printer, Form-2 (Formlabs, Inc.) with Clear FLGPCL04 resin, which was provided by the same company and can provide excellent optical property. The uncured resin was removed by flushing Isopropyl Alcohol and the printed modules were post-cured under a UV light for 30 minutes. These modules are also relatively simple and small which can be printed and post-cured at a time. The whole period required for preparing all of these modules for use in our experiments is about 3 hours. The basic geometry of the T-junction structure is illustrated in Fig. 1c. The vertical conical channel with nozzle diameter *W* = 239 µm introduces the dispersed phase *Q*_*I*_, whereas horizontal square channel with height *H* = 290 µm introduces the continuous phase *Q*_*O*_. They both flow out from the downstream square channel with height *D*_*M*_ = 700 µm.

As shown in Fig. 1a, the PCU and droplet generator are assembled through a connection part. A notch structure designed here together with O-ring can perfectly connect two parts without any liquid leakage. The fasten angle of snap-fit joints has been optimized as 60°, to provide excellent print quality as well as enough sealing force. No liquid leakage has been found in our experiments (up to 4 bar), and this assembly process can be finished in a minute.

Silicone oils (Beijing Hagibis Technology Ltd.) of different viscosities were used as the oil phase (O), whereas water-glycerin (Sinopharm Chemical Reagent Ltd.) mixtures with different viscosities were used as the aqueous phase (W). A 3D-printed resin (Clear FLGPCL04) was used as the solidification phase for microsphere fabrication. RSN-749 resin (CosBond Ltd.), as a surfactant, was used in the oil phase with 0.25% (v/v), and Tween-20 (Beijing Huabo Ltd.), as a surfactant, was used in the aqueous phase with 0.25% (w/v). Liquids viscosities were measured using a viscometer (NDJ-5S, YOKE INSTRUMENT Ltd.), and interfacial tension (σ = 20 mN m^−1^.) was measured using a tensiometer (DCAT 11, Data Physics Corp.). All data are listed in Table 1. Soluble food dyes with different colors (PT. Gunacipta Multirasa Co.) were used in the aqueous phases for a better observation.

**Table 1:** Liquid use in the present work^a^.

Active W/O		Active W/O/W			Passive W/O/W			Microsphere W/O/W		
inner	outer	inner	middle	outer	inner	middle	outer	inner	middle	outer
1	10	1	100	100	1/20	100	100	20	3D-printed resin	100

^a^W: water-glycerin mixture, unit: cP; O: silicone oil, unit: cSt. Interfacial tension σ = 20 mN m^−1^.

The flow was driven and controlled with up to four syringe pumps (LSP02-2A, Longer Precision Pump Co, Ltd). Two solenoid valves (DW10AA and VX240EA, SMC Corp.) and an OB1 pressure controller MK3 (ELVEFLOW) worked corporately under the control of an Arduino Mega 2560 board (arduino.cc) to produce a stable periodic pressure wave as shown in Fig. 1b. The response time of the solenoid valves is 10 ms. The excitation frequency can be achieved up to 30 Hz. Video clips were captured with a high-speed CMOS camera, Phantom VEO 710 L (Vision Research Corp.). The droplet generation was controlled by altering the flow rates of different phases. After reaching the steady state, the generation frequency was monitored and about 50 droplets were analyzed with ImageJ. All experiments were conducted at room temperature (22 °C).

## Results and Discussion

### Effects of the deformation of the flexible channel on the inner flow rate

The droplet generation can be controlled by the variation of the flow rates of different liquid phases, which can be achieved *via* the flexible channel deformation where liquid phases pass through. In our study, the channel deformation is achieved by the pneumatic control in a simple and controllable manner. Therefore, we need to first investigate the effects of the deformation of the flexible channel on the variation of flow rates. Since quite limited studies regarding the multimaterial 3DP technology have been reported so far, some basic printed-material properties are still unknown. We have conducted experiments to measure the Young’s modulus of TangoPlus material for the fabrication of flexible channels, and obtain *E* = 0.504 MPa. The Poisson’s ratio *ν* = 0.495–0.499 is supplied by the provider. Then a numerical model has been built to analyze the deformation of the flexible channel under different applied air pressures. Finally, we can build a relation between the variation of the flow rates and the pneumatic excitation:

1 $$Q(t)={Q}_{I}+{Q}_{A}\,\cos (2\pi {f}_{F}t)$$

The variation of the flow rate *Q(t)* over time *t* can be expressed as the sum of the fixed inner flow rate supply *Q*_*I*_ and the additional flow rate introduced by the excitation. *f*_*F*_ is the excitation frequency of the pressure wave. *Q*_*A*_ is the excitation amplitude of the flow rate, expressed as *Q*_*A*_ = *2πKP/B*, where *K* and *B* are the coefficients determined by the experiments and *P* represents the peak value of the applied pressure wave. All details can be found in the SI (“Experimental and numerical analysis on the deformation of flexible channels” and “Flow rate estimation”). According to equation (1), we can conclude that the total flow rate *Q(t)* depends on the fixed flow rate input *Q*_*I*_ and the flow rate amplitude *Q*_*A*_. Meanwhile, as shown in Fig. S2d, an increase of the applied pressure can increase the additional flow rate. On the other hand, with the constant excitation pressure, the amplitude of the additional flow rate keeps constant while the frequency increases with increasing *f*_*F*_. Furthermore, the positive *Q(t)* corresponds that the liquid is pushed out due to the channel deformation, while the minus *Q(t)* corresponds that the liquid retracts due to the channel back to the normal position. If *Q*_*I*_ is supplied in a small amount, the liquid can be sucked back to the channel. This excitation property can have influence on the droplet generation, which will be studied later.

### Effects of excitation frequency on droplet generation

**Figure 2:**
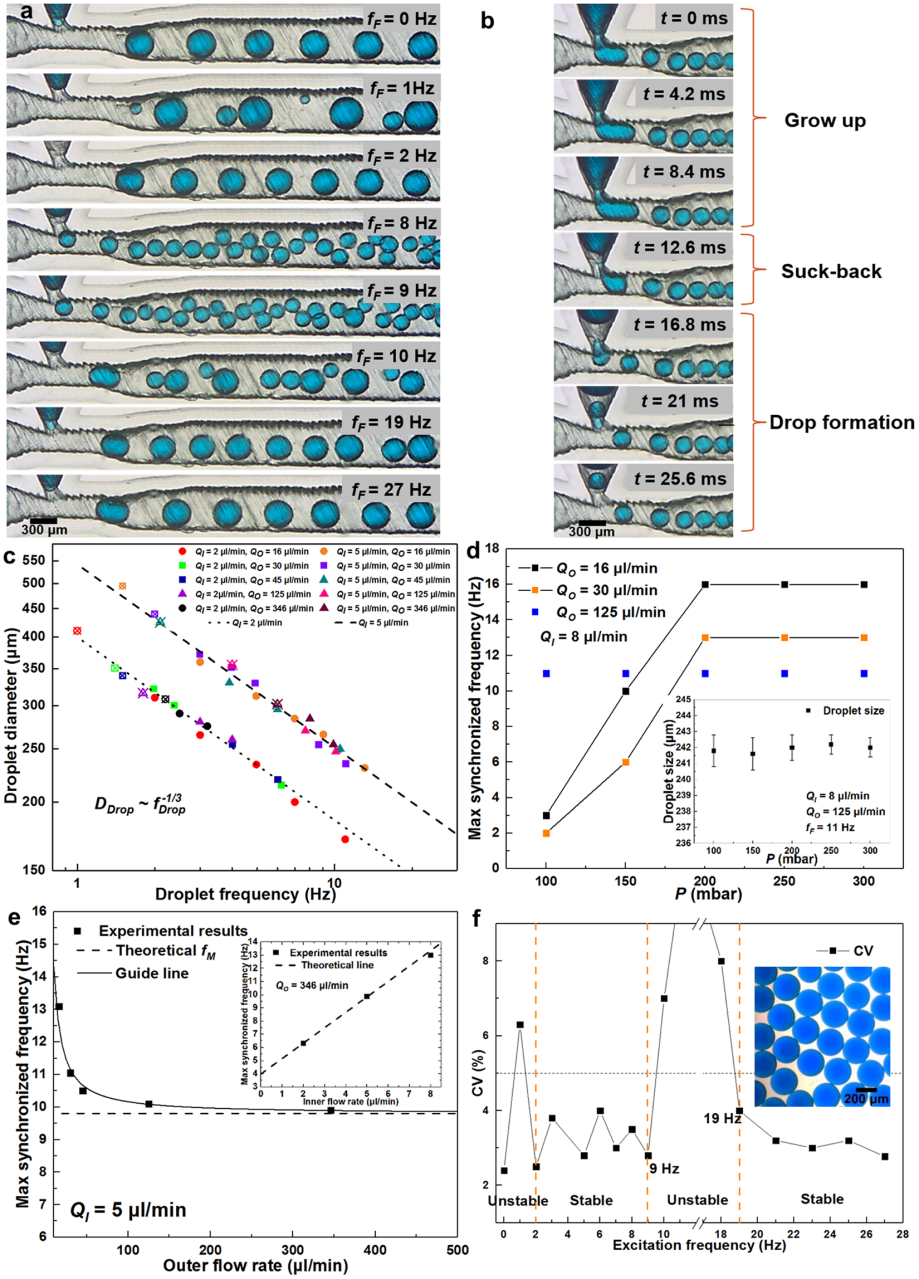
(**a**) High-speed video frames showing the droplet generation with different excitation frequencies. *f*_*F*_ = 0 Hz is the natural state without excitation, *f*_*F*_ = 1 Hz is the unstable state, *f*_*F*_ = 2–9 Hz is the stable synchronized regime, *f*_*F*_ = 10–19 Hz shows the unstable output, and *f*_*F*_ > 19 Hz shows the output of droplets unaffected by the excitation. (**b**) A cycle of the suction-generation process with *f*_*F*_ > 9 Hz. (**c**) Droplet diameter versus droplet generation frequency. P = 200 mbar. Points with cross relate to the natural states without active control. (**d**) Plot of the variation of maximum synchronized frequency with different applied pressures, and the inset showing the droplet size generated in the synchronized regime with different applied pressures. (**e**) Plot of the variation of maximum synchronized frequency with the outer flow rate change, and the inset showing variation of maximum synchronized frequency with the inner flow rate change. P = 200 mbar in both figures. (**f**) Plot of CV value as a function of the excitation frequency.

The dimensions of the channel for droplet emulsion are depicted in the Fig. 1c. *Q*_*I*_ was kept as 5 μl/min. *Q*_*O*_ was kept constant as 16 μl/min. The excitation frequency *f*_*F*_ was varied and the applied pressure was kept constant as 150 mbar. The natural droplet generation frequency without excitation (*f*_0_) is 2 Hz. When the excitation is applied, four regimes have been discovered as shown in Fig. 2a: (1) unstable droplet generation when *f*_*F*_ < *f*_0_ (<2 Hz); (2) stable droplet generation, synchronized with the excitation frequency (2–9 Hz); (3) unstable droplet generation (10–19 Hz); (4) stable droplet generation with the natural generation frequency (>19 Hz). The value of coefficient of variation (CV) of droplet sizes has been calculated in these regimes as depicted in Fig. 2f. The high CV value demonstrates the unstable generation of droplets, while the CV value smaller than 5% is shown in the stable regime. In regime (1), both instability (capillary and excitation instability) are superpositioned and the unstable droplet generation occurs. In regime (2), the excitation effects dominate the droplet generation and monodisperse droplets are produced. In regime (3), the excitation impacts become weak and polydisperse droplets are generated. In regime (4), excitation has negligible influence on the droplet generation, and the droplet generation frequency goes back to the natural generation frequency with narrow size distribution.

Additionally, a new suction-generation regime has been discovered in the synchronized regime. A complete cycle of suction-generation process has been depicted in Fig. 2b. A certain volume of the dispersed phase is first pushed to the junction, and then it retracts in that the flexible channel returns to its normal position and pulls back some of the dispersed phase. This reverse flow pinches off the dispersed phase at the junction which is already surrounded by the continuous oil flow. The droplet size in this regime is always comparable to the junction size, and the drop size distribution is small. This regime can only happen when the excitation frequency *f*_*F*_ is slightly below the maximum synchronized frequency (e.g. 8–9 Hz in Fig. 2a).

In the synchronized regime, the average inner flow rate of *Q(t)* is constant *Q*_*I*_. Based on the conservation law, we have ${Q}_{I}=\frac{1}{6}\pi {D}_{{\rm{Drop}}}^{3}\cdot {f}_{{\rm{Drop}}}$, where *D*_*Drop*_ is the droplet size and *f*_*Drop*_ is the droplet generation frequency. Then we can obtain

2 $${D}_{{Drop}}={(\frac{6{Q}_{I}}{\pi })}^{\frac{1}{3}}\cdot {f}_{{Drop}}^{-\frac{1}{3}}$$

For a fixed *Q*_*I*_, *D*_*Drop*_ scales with *f*_*Drop*_ as ${D}_{{Drop}} \sim {f}_{{Drop}}^{\,-1/3}$. As shown in Fig. 2c, experimental results agree well with the theoretical prediction given by equation (2). We can see that the droplet generation frequency mainly affects the droplet size. Therefore, wider range of droplet size can be produced. The excitation can bring faster pinch-off of the dispersed phase, and even smaller droplet, smaller than the junction size, can be generated with the low flow rate of the dispersed phase, which can benefit for various applications.

### Effects of excitation amplitude and flow rates on the synchronized regime

By integrating *Q(t)* over half of the excitation period [−1/4*f*_*F*_, 1/4*f*_*F*_] during which the dispersed phase protrudes, we can estimate the maximum volume of inner fluid tip *V*_*Tip*_. Using equation (1), we have

3 $${V}_{{Tip}}={\int }_{-1/4{f}_{F}}^{1/4{f}_{F}}{Q}({t})d{t}=\frac{{Q}_{I}}{2{f}_{F}}+\frac{2{KP}}{B{f}_{F}}$$

To form the droplet, *V*_*Tip*_ must be larger than the critical volume *V*_*C*_. Then we have

4 $$\frac{{Q}_{I}}{2{f}_{F}}+\frac{2{KP}}{B{f}_{F}}\ge {V}_{C}$$

As a result, ${f}_{{F}}\le \frac{B{Q}_{I}+4{KP}}{2B{V}_{C}}$, which means the maximum synchronized frequency *f*_*M*_ is

5 $${f}_{M}=\frac{{Q}_{I}}{2{V}_{C}}+\frac{2{KP}}{B{V}_{C}}$$

According to equation (5), we can conclude that if *Q*_*I*_ is increased, the synchronized regime can be extended. The increase of the applied pressure *P* can also increase the range of synchronized regime. It is a little complicated for *V*_*C*_. If *V*_*C*_ is too large (*V*_*Tip*_ << *V*_*C*_), the dispersed phase cannot be pinched off during one excitation cycle and droplets will be formed with several excitation cycles, which results in an unstable regime. If *V*_*C*_ is too small (*V*_*Tip*_ >> *V*_*C*_), the dispersed phase can be pinched off to several droplets during on cycle, which also results in an unstable regime. *V*_*C*_ mainly depends on the Capillary number (${Ca}={\mu }u/\sigma ,\mu$: dynamic viscosity, *u*: characteristic velocity), flow rate ratio $({\varphi }={Q}_{O}/{Q}_{I})$ and channel geometry^14,54^. Increasing *Ca* and *φ* can reduce the value of *V*_*C*_. The minimum *V*_*C*_ is limited by the nozzle diameter *W*, and can be expressed as ${V}_{{C},{\min }}=\pi {W}^{3}/6$.

Now we conduct experiments to verify our analysis. The excitation amplitude is firstly studied. A fixed inner flow rate is given as *Q*_*I*_ = 8 μl/min. The outer phase flow rate *Q*_*O*_ is varied. As shown in the inset of Fig. 2d, the applied pressure has little influence on the droplet size. However, the increase of the applied pressure can extend the maximum synchronized frequency *f*_*M*_, which agrees with our analysis. With the low and moderate flow rate of the outer phase, the influence of applied pressure on *f*_*M*_ is significant. But above 200 mbar, *f*_*M*_ tends to a constant value. The applied pressure over 300mbar may lead to the generation of unpredictable small satellite droplets. With a high outer phase flow rate (125 μl/min), *f*_*M*_ is constant and the applied pressure has no influence. It is obvious that the flow rate ratio has a significant influence on the *f*_*M*_. The flow rate effects will be studied later.

The applied pressure is kept as 200 mbar to investigate the flow rate effects on the *f*_*M*_. First, we keep *Q*_*O*_ = 346 μl/min as constant and vary *Q*_*I*_. In this large outer phase flow rate, *V*_*C*_ should approach its minimum value, ${V}_{C}={{V}}_{{C},{\min }}=\pi {W}^{3}/6$. Therefore, based on equation (5), we have *f*_*M*_ = 1.17*Q*_*I*_ + 3.97. As shown in the inset of Fig. 2e, the experimental results fit very well with our theoretical prediction without fitting parameters. Increasing *Q*_*I*_ can linearly increase the *f*_*M*_. Next, we keep *Q*_*I*_ = 5 μl/min as constant and vary *Q*_*O*_. With the increase of *Q*_*O*_, *f*_*M*_ decreases and tends to a constant value. Again, based on equation (5), we can get *f*_*M*_ = 9.8* Hz*, which agrees well with the experimental results.

### Multiple emulsion generation with 3D-printed modular modules

#### Modular design strategy

Due to the complexity of generation of multiple emulsions which normally include complicated operation of flow rates of different phases, complicated wetting conditions of channel walls and complicated structures, only a couple of studies^33^ have demonstrated that the 3DP technology can be applied for the generation of multiple emulsions. In this section, we are trying to extend 3DP technology applications in both passive and active control of the double emulsion production. One of the advantages of the 3DP technology lies in its capability of modular design and manufacture. Therefore, we design and fabricate the double emulsion generator in a modular way. Modular designs can lower the complexity of the microfluidic chips and also allow for their easy exchange.

**Figure 3:**
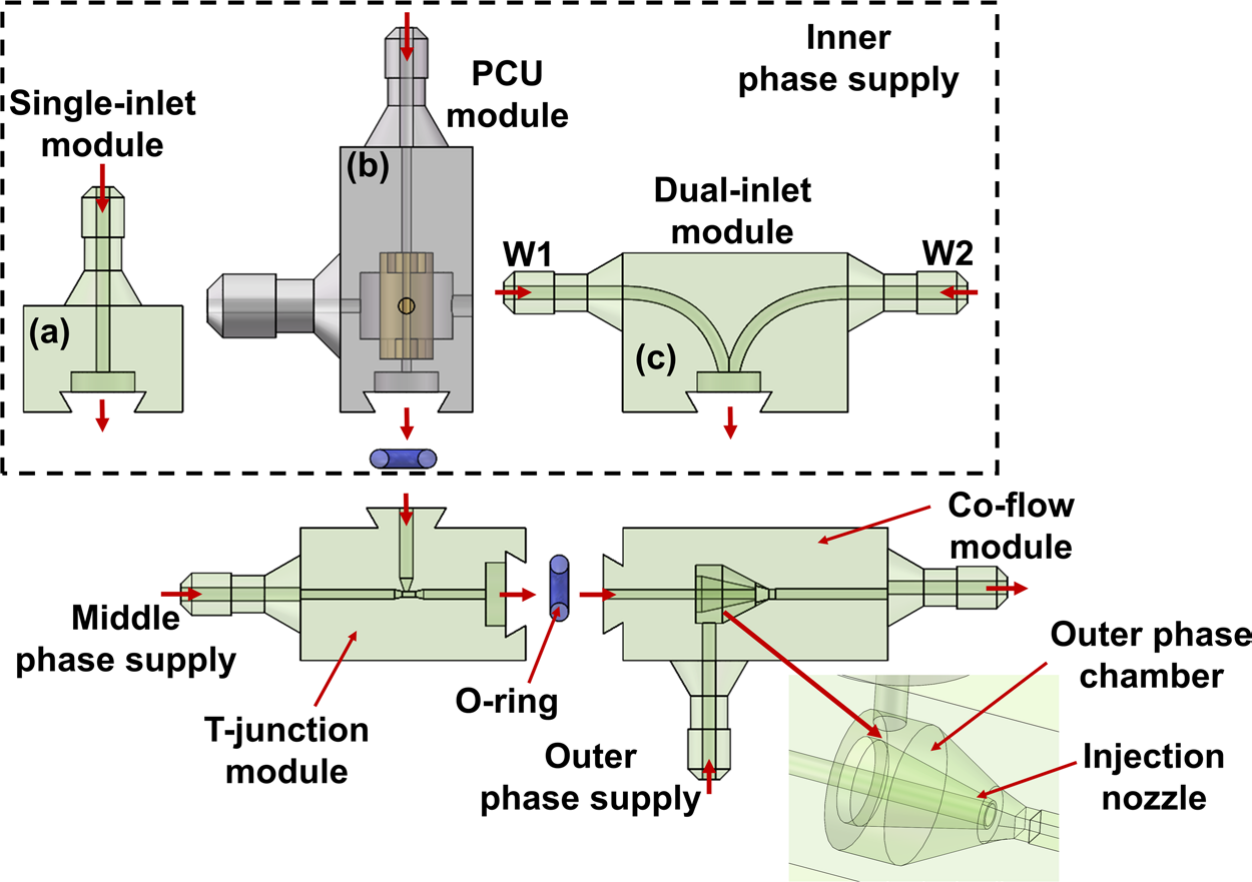
Schematic of the 3D-printed modular device. It consists of three parts: function module, T-junction module, and co-flow module. The function module includes (**a**) Single-inlet module, (**b**) PCU module, (**c**) Dual-inlet module.

As shown in Fig. 3, the emulsion generator includes function module, T-junction module, and co-flow module. Different modules are assembled to each other with snap-fit joints which has been introduced previously. According to different requirements, function module can be chosen as: (1) Single-inlet module for passive generation of double emulsions with different number of the inner droplets. (2) PCU module for active generation of double emulsions with different number of inner droplets. (3) Dual-inlet module for passive generation of double emulsions with different compositions of inner droplets. The single-inlet module, dual-inlet module, T-junction module and co-flow module were printed using the Form-2 printer as mentioned before.

T-junction module is used for the inner droplet generation at upstream, and co-flow module is used for the outer droplet generation at downstream. Co-flow structure was firstly creatively introduced by Weitz’s group through assembly of glass capillaries^18^, but the fragile capillary nozzle and manual operation limit its applications for non-professions. Here we have designed and printed a monolithic co-flow structure. As shown in Fig. 3, a cone structure with a channel inside is printed. The cone chamber is used for outer phase input and also blocking the outer phase to meet the middle phase, and the middle phase reaches the nozzle from the inside channel. If the chip is printed as a whole, this nozzle is difficult to shape due to the difficulty to remove uncured resin through the whole chip. But for a modular design, the uncured resin can be removed through this small co-flow module. Furthermore, modular design and assembly strategy can bring another benefit for the surface treatment. The generation of multiple emulsions usually requires different wetting conditions of channel wall. It is still complicated to obtain different wetting conditions in a single chip, although a couple of methods to solve this issue have been proposed^55–57^. Here, we can easily make different surface treatments for different modules, to achieve the requirements for the generation of multiple emulsions. Different microdevices with different assembled modules (T-junction module and co-flow module are fixed, only function module needs to be changed.) have been demonstrated in the following sections.

#### Active control of double emulsion generation

**Figure 4:**
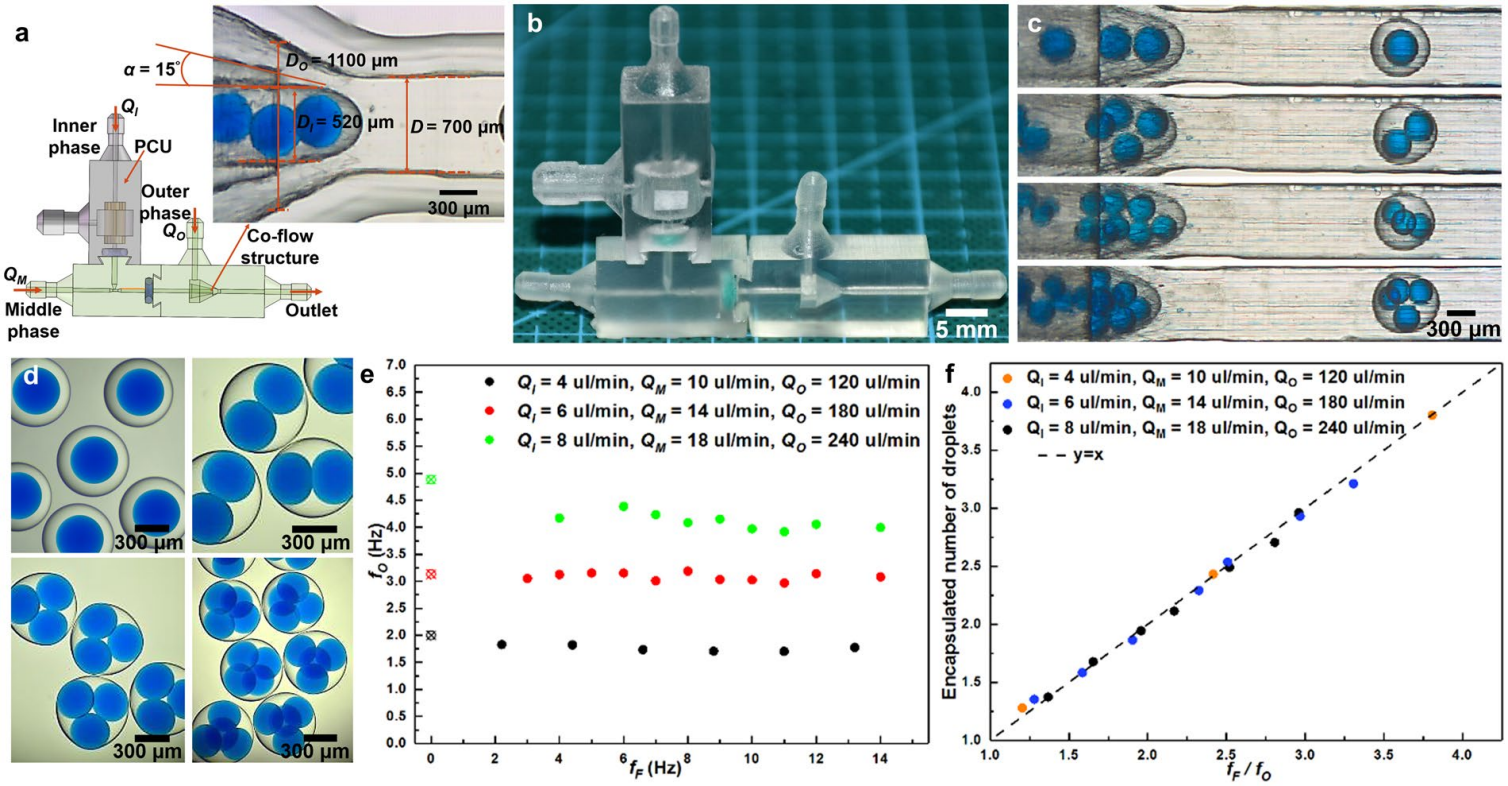
(**a**) Schematic of assembled active control device with PCU module and the illustration of the co-flow structure. (**b**) A printed sample of the assembled active control device. (**c**) High-speed video frames showing active control of W/O/W double emulsions with different encapsulated number of inner droplets. (**d**) Photographs of the different W/O/W double emulsions. (**e**) Plot of the generation frequency of the outer droplet at downstream versus the excitation frequency. Points with cross inside represent natural frequencies. (**f**) Plot of the encapsulated droplet number corresponding to the ratio of the excitation frequency and the generation frequency of outer droplets.

Here PCU module is chosen to be assembled with T-junction and co-flow modules as shown in Fig. 4a,b. *Q*_*I*_, *Q*_*M*_ and *Q*_*O*_ are the inner, middle and outer phase flow rates, respectively. The inner droplets were generated at different frequencies *via* our pneumatic control method which has been fully discussed in the previous sections, and the outer droplets are generated through co-flow structure at downstream. The synchronized regime is used here for double emulsions production. The T-junction channel wall was surface treated as hydrophobic and the co-flow channel wall was surface treated as hydrophilic. W/O/W double emulsions can be produced under this wetting condition. The liquids used for generation of double emulsions are listed in the Table 1. A relatively viscous middle phase used here can reduce the influence of the upstream oscillation on the downstream droplet generation. Alternatively, a longer channel can be used here instead of using viscous middle phase to reduce the upstream influence. As shown in Fig. 4e, different excitation frequency applied at upstream has little influence on the droplet generation at downstream. The number of encapsulated droplets *n* can be simply expressed as:

6 $${n}=\frac{{f}_{I}}{{f}_{O}}=\frac{{f}_{F}}{{f}_{O}}$$

where *f*_*I*_ and *f*_*O*_ represent the droplet generation frequencies of inner and outer droplets, respectively. *f*_*F*_ is the excitation frequency as described previously. Based on the equation (6), double emulsions can be produced with controllable inner droplet number when the inner droplets are generated in the synchronized regime with variation of the excitation frequency. As shown in Fig. 4f, the experimental results have shown an excellent accordance with equation (6). High-speed video frames shown in Fig. 4c have demonstrated that double emulsions with 1–4 of inner droplets are generated with excitation frequency: 3 Hz, 6 Hz, 9 Hz and 12 Hz, respectively. The flow condition is *Q*_*I*_ = 6 μl/min, *Q*_*M*_ = 14 μl/min, and *Q*_*O*_ = 180 μl/min (Also see supplementary movie S1). Microscope photographs shown in Fig. 4d have depicted different double emulsions with different number of inner droplets. We can conclude that generation of double emulsions with controllable inner droplet number can be easily achieved by only altering the excitation frequencies, which avoids the complicated operation of flow rates for different phases to produce different double emulsions.

### Passive generation of double emulsions

#### Single-inlet module for double emulsions

**Figure 5:**
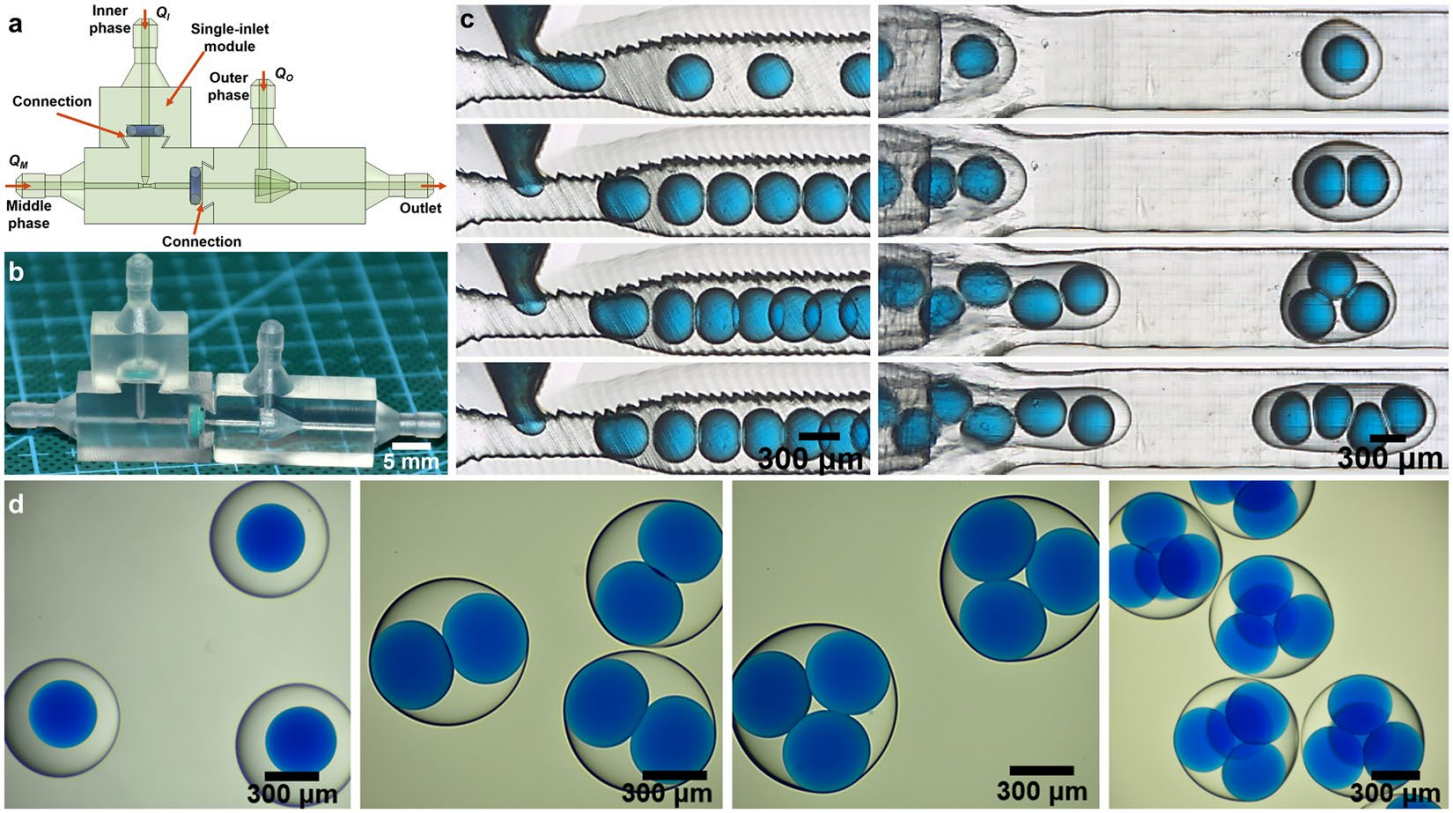
(**a**) Schematic and (**b**) the printed sample of assembled passive double emulsion generation device with single inlet module, respectively. (**c**) High-speed video frames showing the passive generation of W/O/W double emulsions with different number of inner droplets. (**d**) Photographs of different W/O/W double emulsions.

Here single-inlet module is chosen to be assembled with T-junction and co-flow modules as shown in Fig. 5a,b. The same surface treatment was made as described previously, and therefore W/O/W double emulsions can be produced. The liquids used are listed in Table 1. By tuning the flow rates of different phases, double emulsions with different number of encapsulated droplets can be achieved, which is depicted in Fig. 5c (Also see supplementary movie S2). Microscope photographs shown in Fig. 5d have depicted different double emulsions with different number of inner droplets.

#### Dual-inlet module for double emulsions

**Figure 6:**
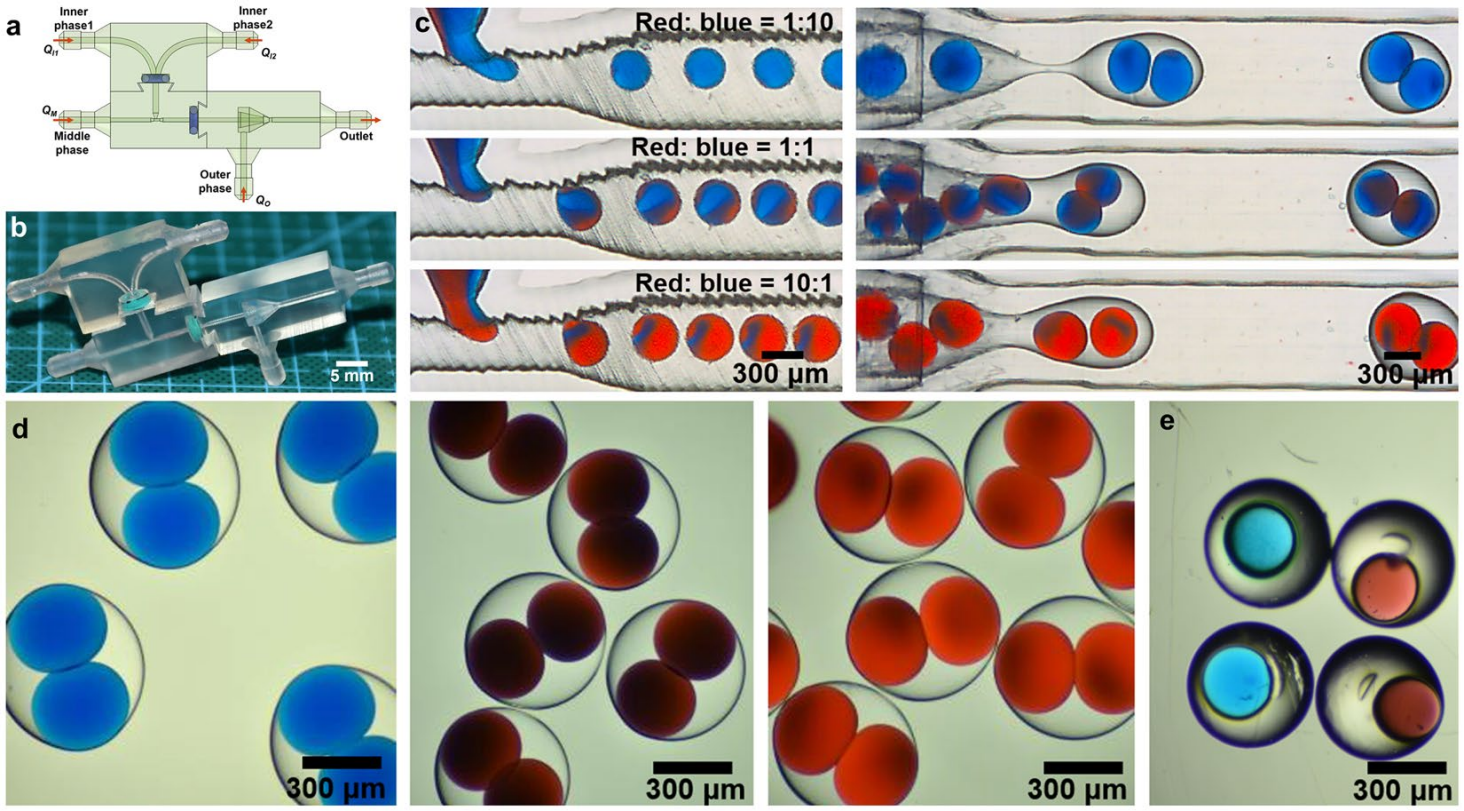
(**a**) and (**b**) is schematic and the printed sample of assembled double emulsion generation device with dual inlet module, respectively. (**c**) High-speed video frames showing generation of W/O/W double emulsions with different compositions of inner droplets. (**d**) Photographs of the W/O/W double emulsion with different compositions of inner droplets. (**e**) Photographs of the microspheres containing different compositions.

Here dual-inlet module is chosen to assemble with T-junction and co-flow modules as shown in Fig. 6a,b. *Q*_*I1*_, *Q*_*I2*_, *Q*_*M*_ and *Q*_*O*_ are the flow rates of the two inner aqueous phases, middle phase and outer phase, respectively. This chip is used for the passive generation of double emulsions with different compositions of inner droplets. The same surface treatment was made as described previously, and therefore W/O/W double emulsions can be produced. Due to the characteristic of the laminar flow in microfluidics, the ratio of two phase volume coming into the droplet depends linearly on the flow rate ratio of two phases as shown in Fig. 6c. Therefore, different compositions of droplets can be acquired accurately after these two phases mix or react inside the droplet by tuning the flow rate ratio. Here we use two inner aqueous phases with different soluble food dyes (red and blue) as a prototyping demonstration. By tuning the flow rate ratio of these two aqueous phases, different compositions of the inner droplets (different mixed colors represent different compositions here) can be achieved, and then the double emulsions encapsulated these droplets are formed at downstream as shown in Fig. 6c (Also see supplementary movie S3). Microscope photographs shown in Fig. 6d have depicted different double emulsions with different compositions. Finally, we use the same device and replace the middle phase with commercial photopolymer as the solidification phase for fabricating functional microspheres containing different compositions which are shown in Fig. 6e. The liquids used are listed in Table 1. Due to the excellent optical properties, transparent microspheres containing droplets with different color can be further applied in optics fields. The structure reported here is painful for other fabrication methods and also these double emulsions with different compositions we produce are difficult to achieve using other methods.

## Conclusions

A PCU device has been designed and fabricated monolithically based on multimaterial 3DP. A 3D coaxial flexible channel is printed by a rubber-like material (TangoPlus FLX930) and other rigid structures are printed by a photopolymer (Veroclear). The numerical results and experiments have demonstrated that this flexible channel can be deformed by the air pressure excitation in a controllable manner. Such deformation can result in additional variation of fluid flow rate when liquids flow through this channel. Therefore, the droplet generation can be actively controlled by tuning the flow rate of the dispersed phase which flow through this flexible channel excited by the air pressure, and then different regimes have been discovered and studied in detail by changing excitation frequency, excitation amplitude and flow rates of both phases. In the synchronized regime, droplet generation frequency fully follows the excitation frequency, and highly monodisperse emulsion droplets can be produced.

Traditional studies^58–60^ usually use polydimethylsiloxane (PDMS) with actuation sources to actively control droplet generation, which requires complicated fabrication process. A monolithic device with different parts can be one-step achieved by multimaterial 3DP technology, which avoids the complicated fabrication process. On the other hand, due to the rigidity of PDMS, a thin PDMS membrane is always required to better translate the actuation. According to our measurement above, the TangoPlus material is softer and therefore thicker flexible channels can be printed, which can ensure excellent translation of actuation and also provide more stability and robustness. Furthermore, a fully 3D coaxial structure can be achieved using multimaterial 3DP technology, which can provide 3D homogeneous deformation for the droplet generation

Furthermore, 3D-printed modular microfluidic devices for multiple emulsion production have been developed, which consist of three modules: function module, T-junction module and co-flow module. The T-junction module is used for the generation of inner droplets, and the co-flow module is used for the generation of outer droplets. The function module can be replaced by single-inlet module, dual-inlet module and PCU module. With assembly of different modules, versatile double emulsions can be produced. By utilizing the synchronized regime abovementioned, double emulsions with different inner number of droplets have been successfully generated without complicated operation of flow rates of different phases. By utilizing the dual-inlet module, double emulsions with inner droplets with different compositions can also be produced successfully. Additionally, transparent microspheres containing different color droplets have been produced, which can be used in the optics field.

We highlight the importance and advantage of our work proposing a modular strategy and combining different 3DP technologies, which can achieve production of versatile complicated multiple emulsions in both active and passive manner by assembly of different modules. This concept and strategy can be further applied in other fields where 3DP is popular such as material engineering and bioengineering. In future, we believe that more complicated devices for microfluidics and more controllable methods for emulsion generation can be developed based on rapidly developing 3DP technologies, especially for multimaterial 3DP.

## Electronic supplementary material

Supplementary Information

Supplementary Movie S1

Supplementary Movie S2

Supplementary Movie S3
