# Supplementary material for: A Modular Microfluidic Device via Multimaterial 3D Printing for Emulsion Generation
Source: Sci Rep. 2018 Mar 19;8:4791. doi: 10.1038/s41598-018-22756-1 (PMC5859176; doi:10.1038/s41598-018-22756-1)
Supplement: Supplementary file 1 — Supplementary Information [file 41598_2018_22756_MOESM1_ESM.pdf]

Supplementary Information

“A Modular Microfluidic Device *via* Multimaterial 3D Printing for Emulsion Generation”

Qinglei Ji[1,2,+], Jia Ming Zhang[2,+], Ying Liu[2], Xiying Li[2], Pengyu Lv[2], Dongping Jin[1], Huiling Duan[2,3,*]

[1]State Key Laboratory of Mechanics and Control of Mechanical Structures, Nanjing University of Aeronautics and Astronautics, 29 Yudao Street, Nanjing 210016, Jiangsu, People’s Republic of China
[2]State Key Laboratory for Turbulence and Complex Systems, Department of Mechanics and Engineering Science, BIC-ESAT, College of Engineering, Peking University, Beijing 100871, People’s Republic of China
[3]CAPT, HEDPS and IFSA Collaborative Innovation Center of MoE, Peking University, Beijing, 100871, People’s Republic of China
[*]hlduan@pku.edu.cn
[+]These authors contributed equally to this work and should be considered co-first authors.

This file includes:
1. Legends of Supplementary Movies S1 to S3
2. Supplementary Information: Experimental and numerical analysis on the deformation of flexible channels.
3. Supplementary Information: Flow rate estimation
4. Supplementary Figures

1. Legends of Supplementary Movies S1 to S3

1.1 Supplementary Movie S1:

Active control of double emulsion generation

The video demonstrates that double emulsions with 1-4 of inner droplets are generated with excitation frequency: 3 Hz, 6 Hz, 9 Hz and 12 Hz, respectively. The flow condition is Q_I = 6 µl/min, Q_M = 14 µl/min, and Q_O = 180 µl/min. The video is recorded with 300 frames per second (fps) and played with 30 fps.

1.2 Supplementary Movie S2:

Double emulsions with different number of encapsulated droplets

This video demonstrates that double emulsions with 1-4 of inner droplets are generated with different flow conditions. The flow conditions are ① Q_I = 2 µl/min, Q_M = 10 µl/min, Q_O = 180 µl/min, ② Q_I = 8 µl/min, Q_M = 14 µl/min, Q_O = 180 µl/min, ③ Q_I = 14 µl/min, Q_M = 16 µl/min, Q_O = 100 µl/min, ④ Q_I = 16 µl/min, Q_M = 16 µl/min, and Q_O = 90 µl/min, respectively. The video is recorded with 300 fps and played with 30 fps.

1.3 Supplementary Movie S3:

Double emulsions with different compositions of encapsulated droplets

This video demonstrates that double emulsions with variable compositions of inner droplets are generated by changing the flow rate ratio of the inner phase while keeping it a constant of Q_I = 15 µl/min. The other flow condition is Q_M = 16 µl/min and Q_O = 160 µl/min. The video is recorded with 300 fps and played with 30 fps.

2. Experimental and numerical analysis on the deformation of flexible channels

15 pieces of specimens (38×7×4.9 mm^3) were printed in total, and 5 specimens for each printing directions: 0° (in the print-line direction), 45° and 90° as shown in Fig. S2a. The strain-stress relation was acquired using a 5940 Series Single Column Table Top Systems (Instron®) with the customized grip and fixture. The maximum strain change that can be applied in our device is about 30%. As shown in Fig. S2a, different printing directions have little influence on the Young’s modulus. Up to the range of 30% strain change, the TangoPlus material has shown a linear strain-stress relation, and still lies in the elastic deformation regime. Such elastic deformation is adequate in our experiments for emulsion generation. The Young’s modulus is therefore calculated as 0.504 MPa. The Poisson’s ratio of TangoPlus material is 0.495-0.499, provided by supplier.

Numerical studies on the deformation of flexible channels were conducted using Abaqus CAE (Dassault Systèmes). As shown in Fig. S2b, uniform pressure is radially applied to the 3D coaxial flexible channel, whose ends both connect to the rigid part. The inner and outer diameters of the flexible channel are 550 µm and 4 mm, respectively, and the flexible channel length is 5mm. These dimensions are in accordance with our actual printed one. For this structure, axisymmetric assumption in vertical direction and symmetry about the horizontal middle plane can be applied, and therefore we can choose 1/8 of flexible channel to make simulations. As shown in Fig. S2b, the upper plane has a fixed support as the boundary condition (BC). The two sides are limited by the axisymmetric condition. BC of the bottom plane is plane symmetry towards the horizontal plane. The additional pressure resulting from the liquid flow in the channel can be negligible due to the small flow rates used in microfluidics for emulsion generation. Simulation results have shown that the center part of channels has the largest radial displacement of about 15 µm under the applied pressure of 200 mbar, and the displacement decreases along the vertical channel. Axial and tangential displacements can be negligible. According to the stability analysis, a critical pressure

over 2 bar will cause the channel buckled and squashed. More than 50,000 cycles with 300 mbar pressure are applied for 10 PCUs, and no one has been found broken or leaked, which has demonstrated that our design and fabrication is robust.

As shown in simulation results (Fig. S2b), when a static pressure is applied, the flexible channel will deform accordingly. The local displacement of the inner channel surface in the radial direction is determined by the pressure P and the axial position z, expressed as $I\left(P,z\right)$. The volume change $V(P)$ caused by this deformation can be achieved through an integration of the displacement over the inner channel surface as shown in equation (S1).

$$V(P)=\int_0^L I(P,z)\pi D_C \mathrm{dz} \quad \text{(S1)}$$

where D_C and L are the inner diameter and length of the channel respectively. The volume change has also been measured by observing the change of liquid meniscus in the glass capillary at downstream. As shown in Fig. S2c, the simulation results agree well with the experimental results. We have also found that the volume change with respect to the applied pressure shows a linear relation, and therefore, the volume change can be expressed with equation (S2).

$$V(P)=KP \quad \text{(S2)}$$

where K = 0.61 nl/mbar is obtained experimentally. It is noteworthy that although the deformation of the flexible channel can be complicated, the volume change resulting from this deformation can be described as a simple linear relation with respect to the applied pressure, which can benefit the engineering applications.

If a periodic pressure $P(t)$ with an excitation frequency f_F is applied, f_F has influence on the deformation of the channel and corresponding volume change (Fig. S2c inset). According to the experimental results, we can express their relation as follows:

$$V(P)=\frac{KP(t)}{1+Bf_F} \quad \text{(S3)}$$

where B = 8.61 s is a fitting coefficient. When $f_F=0$, equation (S3) goes back to equation (S2). If f_F > 1 Hz, $V(P)$ can be simplified as

$$V(P)=\frac{KP(t)}{Bf_F} \quad \text{(S4)}$$

3. Flow rate estimation

The applied pressure waveform has been found to play a minor role in the droplet generation in our experiments, so we present the pressure:

$$P(t)=P\sin(2\pi f_F t) \quad \text{(S5)}$$

By replacing the term P in equation (S4) with equation (S5), we obtain the volume change $V(\mathrm{t})$ with respect to the applied pressure and time t:

$$V(t)=\frac{KP}{Bf_F}\sin(2\pi f_F t) \quad \text{(S6)}$$

The actual flow rate can be described with a sum of the fixed inner flow rate Q_I supplied by a syringe pump, and the additional flow rate, which is the derivative of volume change $V(t)$ over time t, expressed as equation (S7).

$$Q(t)=Q_I+V'(t)=Q_I+Q_A\cos(2\pi f_F t) \quad \text{(S7)}$$

where Q_A is the flow rate amplitude, expressed as $Q_A=2\pi KP/\mathrm{B}$. According to equation (S7), we can conclude that the total flow rate $Q(t)$ depends on the fixed flow rate input Q_I and the flow rate amplitude Q_A. When $Q_I=0$, we can obtain the variation of pure additional flow rate introduced by the pneumatic control with respect to the dimensionless time t/T as shown in Fig. S2d, where $T=1/f_F$, meaning the period for a corresponding excitation frequency.

4. Supplementary Figures

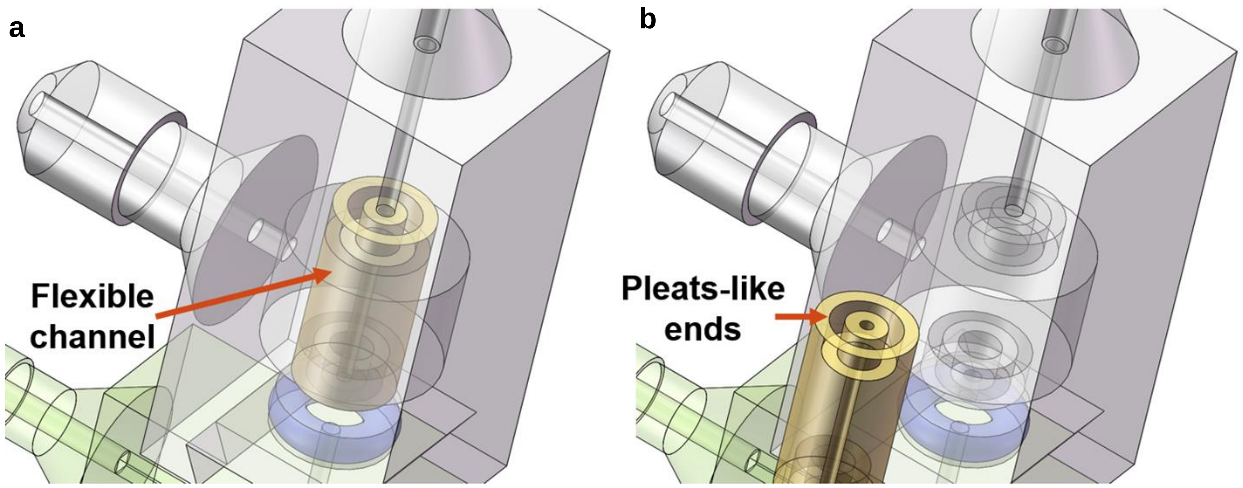

Figure S1. Schematics of the pleats-like ends.

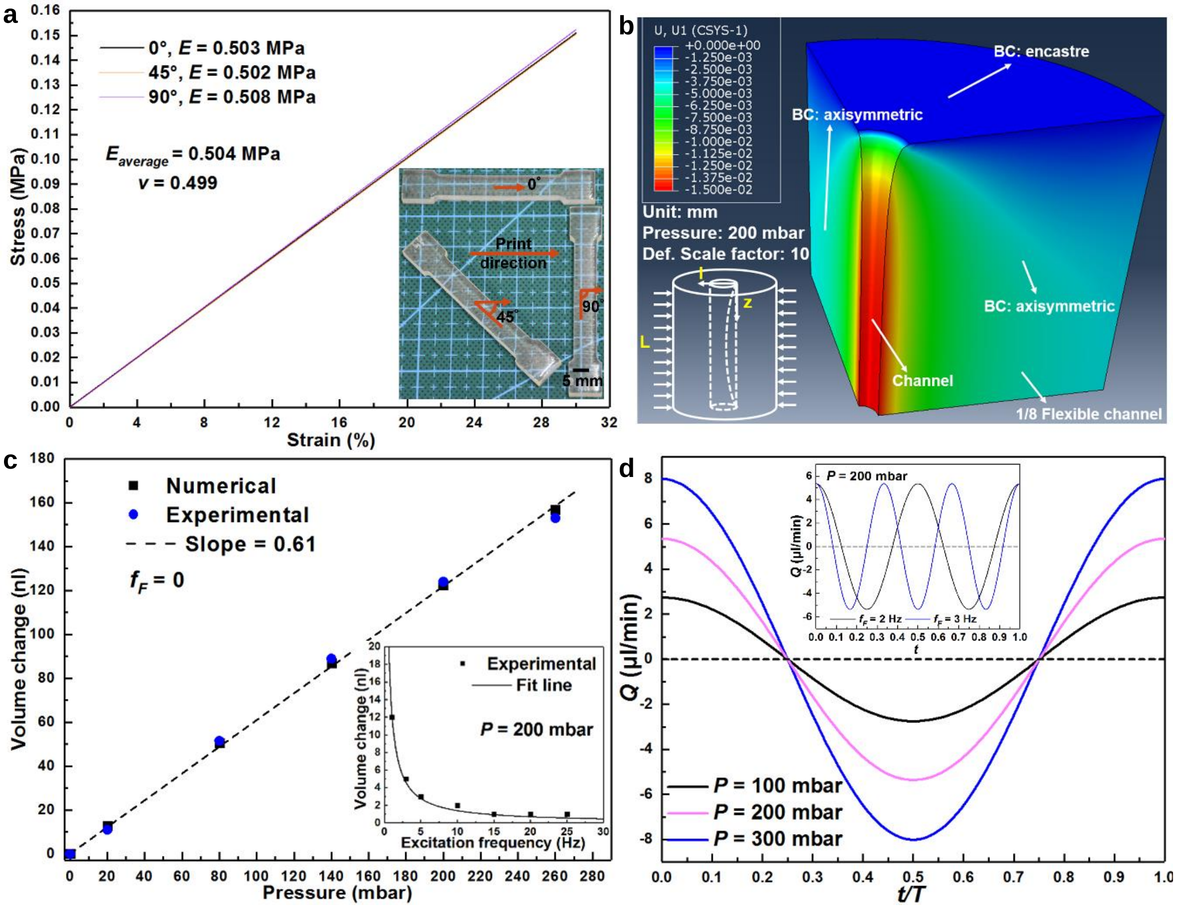

Figure S2. Property of the TangoPlus material and flow rate estimation. (**a**) Stress-strain relation of the TangoPlus material. (**b**) Numerical results for the deformation of the flexible channel under a static applied pressure of 200 mbar. (**c**) Plot of the volume change versus the static applied pressure and the excitation frequency of a periodic pressure. (**d**) The flow rate variation in one excitation cycle with different applied pressures and excitation frequencies.
